# Distinctive Lists of an Insane and Sane Delusion

**Published:** 1878-07

**Authors:** 


Distinctive Lists of an Insane and Sane Delusion. In an indictment for murder, against Alfred Jones, recently tried in the court of Licking county, Ohio, being employed as an expert in regard to the sanity of the defendant, I was led to study somewhat closely what constitutes an insane delusion. The result may be of interest to your readers, some of whom will undoubtedly be called at some future time to testify on the same point.

The circumstances of the case are briefly these: Alfred Jones, aged 72, lived in a secluded dell with an only daughter, aged 42, of irreproachable character. He was a narrow-minded, uneducated man, of good character in every way, except that he was reported to have been violent and abusive to his children, any disobedience on their part exciting him to an immoderate degree. He became possessed with the notion, a year or two ago, that his daughter was unchaste—that she went to church to meet bad men, and when she left the house for any purpose, he always watched her, on the supposition that it was to meet a paramour. On one occasion, on her return from a ravine near the house, he accused her of the suspected vice, and in a paroxysm of fury seized a hatchet, struck at her, which she warded off, then ran from the house, the father pursuing until he overtook her, and struck her repeatedly on the head, until she was dead. He gathered up his effects, put her clothes in order on the spot where she fell, came to Newark and gave himself up to the sheriff to be executed, as he said he had committed an awful crime, and was a law-abiding man. He gave a perfectly truthful account of the unwitnessed killing to the sheriff, as was subsequently verified. The plea of insanity was entered in defense, of which there was no evidence, except in regard to the delusion as to his daughter's chastity. That it was a delusion, either wilful, malicious, or insane, there could be no doubt; all the witnesses testifying that she was never suspected of unchastity, nor had shown any cause for it to any one, save in the imagination of her father, and even he had to admit that he had never seen any overt act on her part. The principal point in the case turned upon this delusion and the manner in which this might affect the premeditation of the act.

In my testimony I gave four points by which a sane delusion may be known from one that is insane. In no work on Medical Jurisprudence have I met with any clear distinction or test as to what constitutes a healthy or sane delusion from one that is insane or diseased. Only by an attentive study of this subject, and from considerable familiarity with monomaniacs, was I enabled to point them out. As they may be of service to other experts, I proceed to give them.

1st. A man must be compared with himself in order to judge aright as to his soundness or unsoundness. In illustration, look at a ruddy-faced active, athletic person, and then at another who is thin, pale and angular. On the face of things, the mind is ready to decide that the former is a healthy man, and the latter a sickly one. Yet if the latter be questioned he may truthfully say, I am perfectly healthy—there is nothing the matter with me; but if the ruddy, athletic person becomes pale, thin and angular, then the judgment that he is not healthy can scarcely fail in correctness. Precisely so is it with the mind. Has it markedly departed from its wonted or former self? Has the disposition suddenly changed, or have life-long traits of character taken a sudden, unaccountable, and new departure? If they have, then that is one of the evidences that the change is a morbid one.

2d. A sane delusion does not grow—does not increase except by increments of evidence; whereas, the insane delusion, particularly in the old, is apt to grow, and wholly irrespective of fostering external circumstances. In other words, the increase of an insane delusion does not depend upon the accumulation of facts, or upon external circumstances for its increment, but upon the morbid condition of the internal nervous structure for its certainty, persistence and intensity.

3d. An insane delusion differs from a sane one in this: the subject of it exhibits more earnestness, vehemence, or exaltation as to it, than on any other subject. When it is touched upon, it is like touching upon the periphery of a diseased nerve. His best friends, those in whom he has the strongest confidence, and the strongest evidences cannot convince him of his error; and whenever he speaks upon the subject pertaining to his delusion, it is with an earnestness, an unreasonable vehemence, which he exhibits on no other subject.

4th. The insane delusion is more persistent than the sane. A man may imagine on one day that he is coming to poverty—that he has lost all his friends, but in a day or two the delusion is dissipated. Not so with the insane delusion. It persists for weeks, months, or years, in variable intensity, and no amount of evidence, no assurances are sufficient to overcome it.

Now, when these are all present in any given case—when they are clearly proven by the evidence, I think there need be no hesitancy in pronouncing the subject insane; diseased in mind, irresponsible for his false belief, and for his actions grounded on that belief.—*Ohio Medical Recorder.*

